# Investigation of the Impact of Saccharides on the Relative Activity of Trypsin and Catalase after Droplet and Spray Drying

**DOI:** 10.3390/pharmaceutics15102504

**Published:** 2023-10-21

**Authors:** Johanna Dieplinger, Christina Moser, Gerhard König, Joana T. Pinto, Amrit Paudel

**Affiliations:** 1 Research Center for Pharmaceutical Engineering GmbH, 8010 Graz, Austria; johanna.dieplinger@rcpe.at (J.D.); gerhard.koenig@rcpe.at (G.K.); 2 Institute of Process and Particle Engineering, Technical University of Graz, 8010 Graz, Austria; joana.pinto@rcpe.at

**Keywords:** saccharide, cyclodextrin, activity, droplet drying, spray drying, molecular modeling

## Abstract

While using saccharides as stabilizers for therapeutic protein drying is common, the mechanisms underlying the stabilization during drying remain largely unexplored. Herein, we investigated the effect of different saccharides, trehalose dihydrate (TD), dextran (DEX), and hydroxypropyl β-cyclodextrins (low substitution—HP and high substitution—HPB), on the relative activities of the enzymes trypsin and catalase during miniaturized drying (MD) or spray drying (SD). For trypsin, the presence of saccharides, especially HP, was beneficial, as it significantly improved the enzyme activity following MD. The HPB preserved trypsin’s activity during MD and SD. Adding saccharides during MD did not show a notable improvement in catalase activities. Increasing TD was beneficial during the SD of catalase, as indicated by significantly increased activity. Molecular docking and molecular dynamics simulations oftrypsin with HP or HPB revealed the influence of their substitution on the binding affinity for the enzyme. A higher affinity of HP to bind trypsin and itself was observed during simulations. Experimentally, activity reduction was mainly observed during MD, attributable to the higher droplet temperature during MD than during SD. The activities from the experiments and aggregation propensity from molecular modeling helped elucidate the impact of the size of protein and saccharides on preserving the activity during drying.

## 1. Introduction

Freeze drying is the primary technique to produce solid biopharmaceuticals, and its market size is predicted to more than double from 2023 to 2031 for freeze-dried biological products, with the Asia-Pacific region making up the largest [1]. Alternatively, spray drying (SD) as a biopharmaceutical production technique is gaining interest as a one-step and rapid drying process [2]. The SD process involves atomizing the feed solution into tiny droplets, followed by transient drying by a hot gas stream to generate dried particles. The SD process also offers the opportunity for continuous and energy-efficient manufacturing of biotherapeutics [3,4,5]. To prevent protein aggregation, denaturation, and activity loss during SD, excipients such as amino acids or saccharides are used [2,6,7,8,9]. Water replacement and vitrification theories are the two reported stabilization mechanisms of saccharides during protein drying [3,10,11,12]. Nonetheless, it is still unclear at what concentration these different saccharides manage to stabilize proteins during evaporative drying, such as SD, successfully. Biopharmaceuticals are relatively expensive and only available in limited amounts during product development. Therefore, miniaturized drying (MD) approaches that allow for quickly generating a substantial amount of data using small sample amounts are desirable. Such approaches allow the determination of droplet drying kinetics and provide additional information on the evaporation rate as the droplets are larger and the drying times are much longer than with SD. The available methods for MD-based formulation screening belong to film casting or single droplet drying [13,14,15]. The benefits and shortcomings of several MD techniques have been summarized elsewhere [16]. Previously, we systematically screened for the suitability of excipients for the protein SD experiments by applying our straightforward in-house-developed MD method [16]. Presenting a “worst-case” scenario, our MD method can support the selection of promising saccharides for larger-scale industrial SD processes. This means that saccharides, which were found to protect proteins during MD successfully, will also protect them during SD. For the pharmaceutical industry, activity and chemical/physical stability (e.g., aggregation, degradation) during the development of large molecules are two important properties. However, here, we focus on evaluating the relative activity of the proteins, applying our previously published MD approach to evaluate the effect of saccharides on the enzymes trypsin and catalase during MD and SD. These enzymes have been selected based on their size differences to gain deeper insight into the relationship between the stabilization propensity and the sizes of different proteins and saccharides. Therefore, differently-sized saccharides such as trehalose dihydrate (TD), dextran (DEX), and two differently substituted cyclodextrins (CD), namely hydroxypropyl β-cyclodextrins (HPB and HP), were applied. To corroborate the impact of CD chemistry on the activities of these enzymes, molecular modeling was performed.

## 2. Materials and Methods

### 2.1. Materials

The enzymes trypsin from bovine pancreas [17] with a size of 23.8 kDa (Sigma Aldrich, St. Louis, MO, USA; ≥25,000 units/mg; PDB:7BRX) and tetrameric catalase from bovine liver [18], having a size of 250 kDa (BOC Sciences, United States; 2000–5000 units/mg; PDB:1TGU), were chosen for this study. Trehalose dihydrate (named ‘TD’), a disaccharide (Merck KGaA, Darmstadt, Germany; Mw = 378.33 g/mol), and dextran 40 EP (named ‘DEX’), a polysaccharide (Pharmacosmos A/S, Holbæk, Denmark; Mw = 40,000 g/mol) were used. Two differently substituted hydroxypropyl β-cyclodextrins (HPβCD) were used as oligosaccharides (named ‘HP’ and ‘HPB’). The higher substituted HP (Kleptose^®^ HP ORAL GRADE, Roquette Frères, Lestrem, France; Mw = 1501 g/mol; nominal molar substitution = 0.9) and the lower substituted HPB (Kleptose^®^ HPB, Roquette Frères, France; Mw = 1387 g/mol; nominal molar substitution = 0.62) were used. Different enzyme-saccharide formulations were prepared, which contain only one enzyme and one saccharide at a time (Table 1). Purified water was used to prepare the formulations (TKA Wasseraufbereitungssysteme GmbH, Niederelbert, Germany). The applied concentrations of enzymes and saccharides were based on our previous study [16]. The enzyme concentrations were fixed at around 10 wt.% for each formulation, corresponding to 0.4 mM for catalase and 4.2 mM for trypsin. Different chemicals purchased from Sigma Aldrich (St. Louis, MI, USA), such as Nα-benzoyl-L-arginine ethyl ester hydrochloride (BAEE), sodium phosphate monobasic monohydrate, and potassium phosphate dibasic trihydrate, were used for the activity assays. Sodium hydroxide was purchased from Thermo Fisher Scientific (Waltham, MA, USA). 30 wt.% hydrogen peroxide was purchased from VWR Chemicals (Karlsruhe, Germany), whereas hydrochloric acid and potassium dihydrogen phosphate were purchased from Roth (Karlsruhe, Germany) and Merck (Darmstadt, Germany), respectively.

### 2.2. Miniaturized Drying (MD)

The miniaturized drying approach (named ‘MD’) is described in detail in our previous work [16]. Briefly, droplets with a volume of 15 µL were dispensed onto a polypropylene film attached to a Teflon membrane located on a hot plate (Hei-Standard magnetic stirrer, Heidolph Instruments GmbH & Co. KG, Schwabach, Germany). The hot plate temperature was 69.7–71.8 °C, and the membrane temperature with the polypropylene film attached was 62.0–67.1 °C. The appropriate amount of each enzyme was weighed and dissolved in purified water without or with the correct amount of saccharide stock solutions. The required volumes were added on a scale using Eppendorf pipettes. Only one type of enzyme and saccharide were mixed by pipetting up and down and vortexing at medium speed (~1500 rpm) for ~15 s. The saccharides were added to the enzyme formulations at a high ratio (HR), medium ratio (MR), or low ratio (LR). For MD, the EasyDrop setup (EasyDrop, Krüss GmbH, Hamburg, Germany) was used to monitor the single droplet drying process. The equipment used Drop Shape Analysis (DSA1v1.92, Krüss GmbH, Hamburg, Germany) software to visualize the droplets. Figure 2 of Ref. [16] shows the principle of the drying setup. To generate 15 μL droplets, a tip with a 1.8 mm diameter was selected, and 1× zoom was used as magnification. A monochrome CCD camera, coupled with a halogen light source, was part of the EasyDrop equipment. At minute intervals during 15 min of drying per droplet, pictures of the droplets were generated. This enabled us to monitor how the droplet diameter changed over time. Until final analysis, the dried droplets were scratched off the pp-film and frozen.

#### Evaporation Rate and Aspect Ratio

The rate of drying (evaporation rate) was calculated using Equation (1) [19]:

(1) $$d^{2}\left(t\right)=d_{0}^{2}-kt$$

where d is the droplet diameter monitored over time, $d_{0}$ is the initial diameter of the droplet, k is the evaporation rate, and t is the drying time. Based on the generated droplet pictures and for simplicity, we decided to assume the shape of a spherical cap. The droplet pictures generated during droplet drying allowed us to measure the radius and height of the droplets using straightforward measurements in PowerPoint software (Version 2108). By plotting the droplet radius over time, the evaporation rate k was determined as the slope of the linear equation y = k × x + d. This procedure was performed for each droplet (in duplicates), resulting in an average value of k. Additionally, the aspect ratio (AR) after drying the droplets for 15 min was approximated according to Equation (2):

(2) $$AR=\frac{w}{h}$$

Here, *h* is the height and *w* is the width of the dried droplet. The AR gives information on the sphericity, meaning the closer to 1, the more spherical, and the closer to 0, the more needle-like droplet shapes.

### 2.3. Spray Drying (SD)

Based on the results from the MD, six different formulations (*V* = 5 mL) containing a fixed enzyme content were spray-dried. Using trypsin, three formulations with either no saccharide, 75.6 mM HP, or 75.6 mM HPB were spray-dried, whereas using catalase, three formulations with either no saccharide, 200 mM TD, or 400 mM TD were spray-dried (see Table 1). The formulations were prepared as mentioned under 2.1, using ~10 wt.% of trypsin and catalase, and were SD at lab scale (4M8-TriX, ProCepT, Zele, Belgium) using air as the drying gas. The drying chamber was 0.6 m high, and the orifice of the bi-fluid nozzle was 0.6 mm. The following parameters were fixed for all SD runs: air speed (0.8 m^3^/min), cyclone airflow (180 L/min), nozzle pressure (0.6 bar), atomization airflow (8.6 L/min), inlet temperature (110 °C), and feed rate (~1.6 g/min). The maximum droplet temperature of 63 ± 2 °C measured during MD was targeted to be reached as the outlet temperature during SD. After SD, the powders were kept at the outlet conditions for 15 min, so the powders experienced a hot environment for the same time during MD and SD. The produced powders were frozen (−25 °C to 0 °C) until analysis.

#### Determination of Moisture Content

Using the Karl-Fischer titration (SI Analytics Titrator TitroLine^®^ 7500 KF, Xylem Analytics Germany Sales GmbH & Co. KG, Weilheim, Germany), the amount of residual water in the powders after SD (*n* = 3) was determined. It was performed at 21.5 °C room temperature and 58.8% relative humidity. Approximately 50 mg of powder was placed into the titrator cell, and the water was extracted with methanol (Aquastar^®^ CombiMethanol, Merck KGaA, Darmstadt, Germany) for 1 h and quantified.

### 2.4. Molecular Docking and Molecular Dynamics Simulations

The program Molecular Operating Environment (MOE) [20] was used with the default Amber10-EHT force field. Possible ways for Bovine Pancreatic Trypsin (PDB: 7BRX [21]) to aggregate were explored with the Protein-Protein Dock module of MOE and ranked according to their S score. Molecular dynamics simulations were performed with the AMBER22 package [22]. The CHARMM36 force field [23] was used for proteins, while the CHARMM General Force Field [24] was used for cyclodextrins. Three kinds of simulations were prepared: (a) trypsin in an aqueous solution, (b) trypsin with the nine non-overlapping lowest-free energy binding cyclodextrin positions of a β-cyclodextrin with four 2-hydroxypropyl groups linked to the O6 atom of glucose units 1, 3, 5, and 7 (Tet6-HPβCD, now referred to as ‘HPB-LIKE’), (c) trypsin with the nine non-overlapping lowest free-energy binding cyclodextrin positions of a β-cyclodextrin with 2-hydroxypropyl groups linked to the O6 atom of all seven glucose units (Hep6-HPβCD, now referred to as ‘HP-LIKE’). The structures of all simulations were prepared with CHARMM-GUI [25,26], surrounding them with 12 Å of TIP3P water [27], and using a buffer of 0.1 M potassium chloride. The simulations were performed at 42 °C and 63 °C, as these were the lowest and highest temperatures observed during the MD experiments. For trypsin dimer simulations at 315.15 K (42 °C) and 336.15 K (63 °C), two of such individual simulation boxes were assembled via coordinate translations into one larger rectangular box, yielding boxes of 72.4 × 72.4 × 144.8 Å and 72.9 × 72.9 × 145.8 Å, respectively. For the multimer simulations of each type, eight such individual simulation boxes were assembled via coordinate translations into one larger cubic box, yielding sizes between 114.4 Å and 122.4 Å. This corresponds to a ca. 7.2–8.8 mM protein concentration and cyclodextrin concentrations between 64.8 and 79.2 mM (which corresponds to the HR experimental conditions).

### 2.5. Characterization of the Enzymatic Activity of the MD and SD Samples

The enzymatic activities of trypsin and catalase, after drying, were analyzed according to the assay protocols kindly provided by Merck online [28,29]. The protocol for catalase was EC1.11.1.6, and the one for trypsin was EC3.4.21.4. All solutions have been prepared according to these protocols. For our purposes, these assays have been modified accordingly. More details are provided in the next sections. Using the spectrophotometer UV-Vis 2700 (Shimadzu Europa GmbH, Duisburg, Germany) at 25 °C, the different absorbances have been detected (*n* = 3). For each formulation, two dried droplets have been analyzed.

#### 2.5.1. Enzymatic Activity of Catalase Using UV-Vis Spectroscopic Analysis

The 50 mM potassium phosphate buffer at pH 7.0 and the 0.036 wt.% hydrogen peroxide solution (H_2_O_2_) have been prepared according to the protocol from Merck [29]. The absorbance of the H_2_O_2_ solution measured at A_240_ (240 nm) was at 0.547 absorbance units and, therefore, in the required range of 0.520 to 0.550. For example, drying 15 µL of catalase droplets during MD resulted in ~1.5 mg of dried catalase. Hence, by pipetting up and down and vortexing at 2500 rpm for 5 s, the dried droplets were dissolved in 150 µL of the same buffer to produce a concentration of ~10 mg/mL. As mentioned above, the enzyme solutions were diluted to the final 100 units/mL before analysis. For the catalase powders obtained during SD, 10 mg of powder were dissolved in 1 mL of the same buffer (~10 mg/mL). Again, right before the measurements, the secondary dilution to 100 units/mL was performed. After the final dilution of the samples according to the provided assay protocols, they were measured in quartz cuvettes immediately after preparation. For the duration of 180 s using the kinetics mode, the change in the absorbance at A_240_ of H_2_O_2_ (Δ absorbance) was recorded at each second and plotted against the time. The Δ absorbance of the original catalase powder before drying (Raw) served as the reference with 100% activity, to which the activities of all samples were compared. The samples’ relative activity was calculated accordingly, and the sample buffer was used as a blank.

#### 2.5.2. Enzymatic Activity of Trypsin Using UV-Vis Spectroscopy Analysis

The 67 mM sodium phosphate buffer at pH 7.6, the 0.25 mM Nα-benzoyl-L-arginine ethyl ester substrate solution, and a 1 mM hydrochloric acid solution have been prepared according to the protocol from Merck [28]. The trypsin solution had the desired concentration of 425–575 units/mL and was prepared fresh, just before analysis, in the suitable solvent. The dried droplets of the 15 µL initial volume produced by MD contained ~1.5 mg of trypsin. To achieve the desired concentration of ~10 mg/mL, each droplet was dissolved in 150 µL of solution by pipetting up and down and vortexing at 2500 rpm for 5 s. A final concentration of 500 units of trypsin per mL was achieved by further diluting the solution, lying within the desired range given above. For the trypsin powders obtained during SD, 10 mg of powder was dissolved in 1 mL of the same solution (~10 mg/mL). Again, right before the measurements, the secondary dilution to 500 units of trypsin per mL was performed. After the final dilution of the samples according to the provided assay protocols, they were measured in quartz cuvettes immediately after preparation. For the duration of 70 s using the kinetics mode, the increase in absorbance at A_253_ (Δ absorbance) was recorded at each second and plotted against the time. The Δ absorbance of the original trypsin powder before drying (Raw) served as the reference with 100% activity, to which the activities of all other trypsin samples were compared. The relative activity of the samples was calculated, and the sample buffer was used as a blank.

#### 2.5.3. Statistical Analysis and Generation of Graphs

All presented figures in the Results and Discussion sections have been created using the Paired Comparison Plot and the Bonferroni Mean Comparison Method of the OriginPro software, Version 2021 (OriginLab Corporation, Northampton, MA, USA).

## 3. Results

### 3.1. Drying of Catalase Formulations

#### 3.1.1. Formation of Catalase Particles during MD

**Figure 1:**
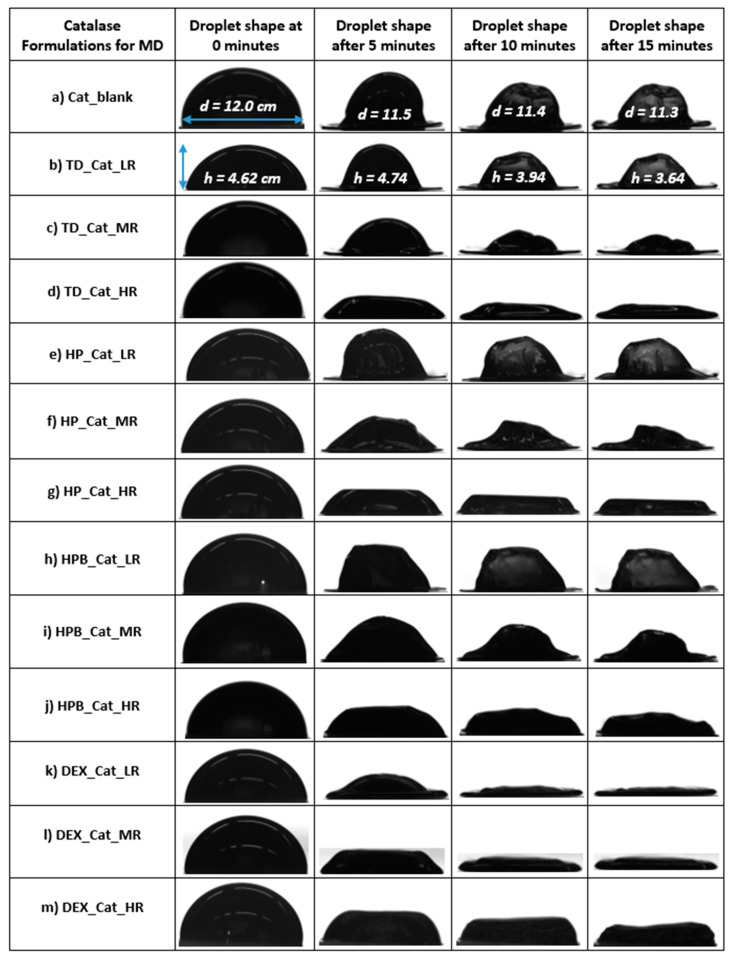
(**a**–**m**): The development of droplet morphology during MD for 15 min of different formulations containing the blank with pure catalase without saccharides (**a**) and catalase with different types of saccharides (**b**–**m**) are presented. Although pictures were taken at minute intervals, only selected time points are shown for simplicity. The values for the determined diameters and heights (in cm) are shown as exemplary for some of the droplets only. MD = miniaturized drying, HR = high ratio, MR = medium ratio, LR = low ratio.

**Figure 2:**
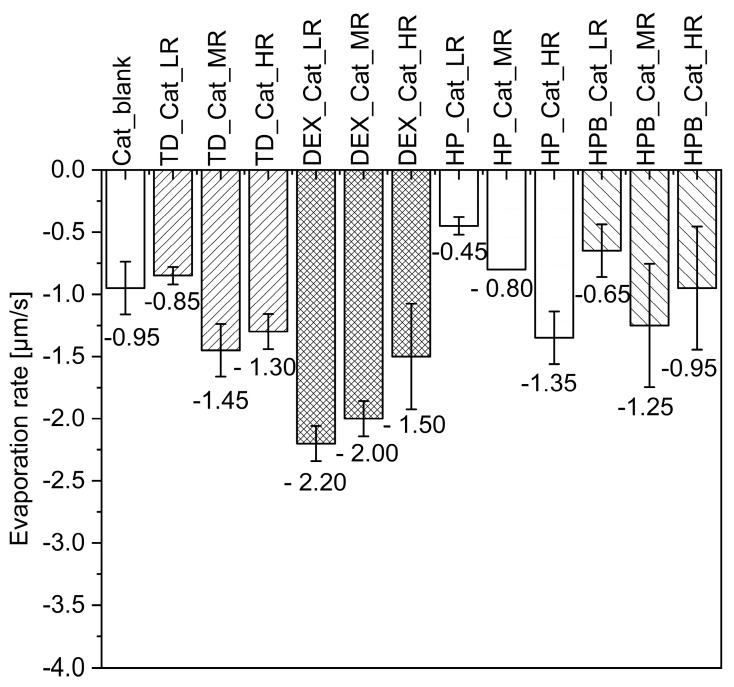
The evaporation rates of catalasesaccharide droplets after MD for 15 min are presented. The blank corresponds to catalase in water without saccharides. The measurements were performed in duplicates, and the bars show the mean value of the evaporation rates ± range (*n* = 2).

Figure 1 summarizes the different shapes of the dried droplets containing catalase alone (a) or catalase and one saccharide (b–m). Figure 1a,b,e,h show the typical “hat shape” morphology obtained for droplets of dried protein formulations [30]. According to Larson et al., flow features could be connected to the droplet shape. They report that the flow, which is directed downwards and outwards, is caused by the sphericity of the droplet cap and the contact line fixed on a chosen surface. The solvent evaporates during droplet drying. However, due to the flow direction, the evaporated solvent at the droplet margin must be substituted with solvent from the droplet core. Due to the tendency of the solvent to move to the droplet surface, Marangoni flow is induced, leading to a higher concentration of proteins at the rim [30]. The authors call this a “Mexican hat” due to the upward buckling of the droplet [30]. Throughout reducing the saccharide while keeping the protein content the same (solid content ~10.1–11.9 wt.%), the mentioned “hat shape” was always restored at LR, with DEX as the only exception (Figure 1k,m). When the saccharide amount was increased, overall flatter droplets were generated, again with DEX being an exception. Similar results were observed in our previous work for protein X [16]. Figure 2 shows the evaporation rates of the dried droplets containing catalase alone or with saccharides. As the content of TD, DEX, and HPB was increased from MR to HR, the evaporation rates decreased, except for droplets containing HP (Figure 2), for which they increased.

Applied at LR, however, all saccharides showed the lowest evaporation rates, which increased from LR to MR, except for DEX, which resulted in the highest evaporation rate at LR (−2.20 µm/s) that decreased from LR to HR (Figure 2). The overall lowest evaporation rate was observed for HP at LR (−0.45 µm/s). In contrast, the highest evaporation rates were observed for all DEX samples (from −2.20 µm/s at LR to −2.00 µm/s at MR to −1.50 µm/s at HR). For TD and HPB, the lowest evaporation rates were observed at LR, while the highest were observed at MR. The following formulations showed lower or higher evaporation rates during MD: HP at LR (−0.45 µm/s) < HPB at LR (−0.65 µm/s) < HP at MR (−0.80 µm/s) < TD at LR (−0.85 µm/s) < HPB at HR (−0.95 µm/s) = blank (−0.95 µm/s). As shown in Supplementary Figure S1, the AR for dried catalase droplets containing DEX was lower with lower DEX ratios. This aligns with the observations that dried catalase droplets with DEX were generally relatively flat (Figure 1k,m). The highest AR was observed with HP at a low ratio, resulting in a hat shape (Figure 1e). 

#### 3.1.2. Catalase in-Process Activity during MD

**Figure 3:**
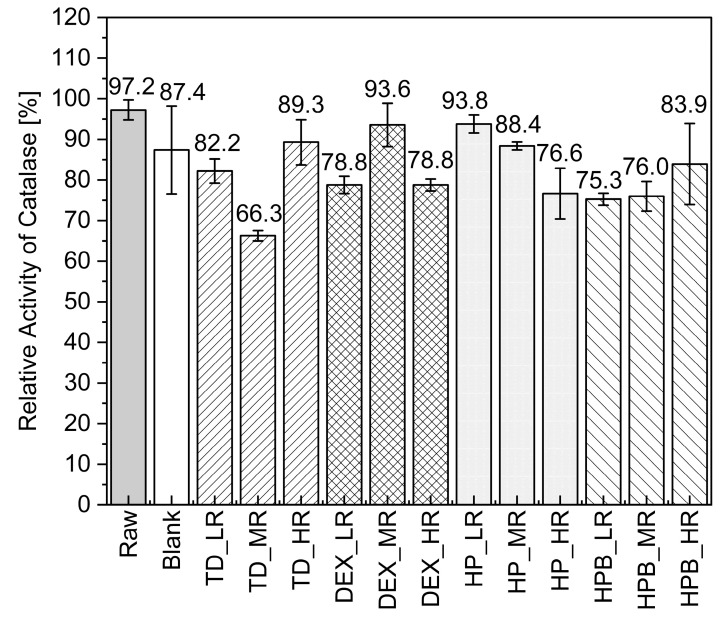
Relative activities of catalase-saccharide droplets after MD for 15 min. The blank is catalase in water dried without saccharides. The reference raw is the undried raw material. The bars present the mean relative activities ± error evaluated from dried droplets (doublets), analyzed in triplicate (*n* = 6).

Drying catalase droplets without adding saccharides (Blank) did not lead to a loss in activity. The relative activities obtained after MD of the different catalase formulations with and without saccharides are collectively shown in Figure 3. For TD-containing formulations, no clear trend could be observed. Surprisingly, TD at MR even notably decreased catalase activity (66.3%), although this disaccharide is well-known to be very good at protecting proteins during drying [2].

None of the dried TD formulations showed significantly improved activities than the catalase dried alone (87.4%). When TD was used at HR, slightly higher activity was observed (89.3%) than at LR (82.2%). Four formulations showed higher activities than the blank dried without saccharides (87.4%), namely DEX at MR (93.6%), HP at LR (93.8%), TD at HR (89.3%), and HP at MR (88.4%). For the droplets containing HP, the activity decreased as the ratio increased from LR (93.6%) to HR (76.6%). In contrast, for HPB, the activity increased as the ratio increased from LR (75.3%) to HR (83.9%). The opposite behavior of HP and HPB was observed with their ratios and resulting catalase activity.

#### 3.1.3. Formation of Catalase Particles and Activity during SD

The results of the Karl Fischer titration of the spray-dried powders are as follows: the highest water content of 8.11 ± 0.28% was observed for catalase samples SD without saccharides. When the saccharide TD was present, lower water contents of 4.47 ± 0.24% (for TD at HR) and 6.17 ± 0.13% (for TD at MR) were observed. Hence, of the three spray-dried powders, TD at HR led to the lowest moisture content, corresponding to a reduction of ~44% compared to catalase SD without saccharides. Significantly higher activities were observed at HR when comparing TD at MR and HR (Figure S2A). In Figure S2B, after SD, a significant increase (*p* ≤ 0.001) of catalase activity for the formulations containing TD (113% at MR and 119% at HR compared to 92.9% for catalase alone) was observed. This indicates that adding TD to catalase benefits the enzyme activity during SD.

### 3.2. Drying of Trypsin Formulations

#### 3.2.1. Formation of Trypsin Particles during MD

**Figure 4:**
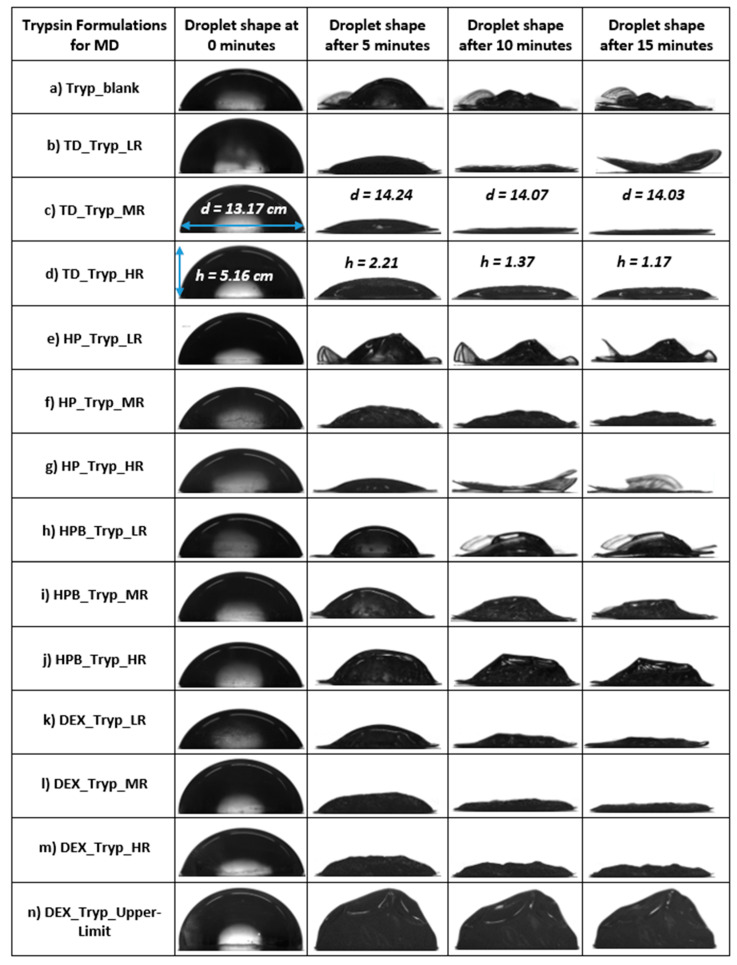
(**a**–**n**): The development of droplet morphology during MD for 15 min of different formulations containing the blank with pure trypsin without saccharides (**a**) and trypsin with different types of saccharides (**b**–**m**) are presented. Although pictures were taken at minute intervals, only selected time points are shown for simplicity. The values for the determined diameters and heights (in cm) are shown as exemplary for some of the droplets only. MD = miniaturized drying, HR = high ratio, MR = medium ratio, LR = low ratio.

**Figure 5:**
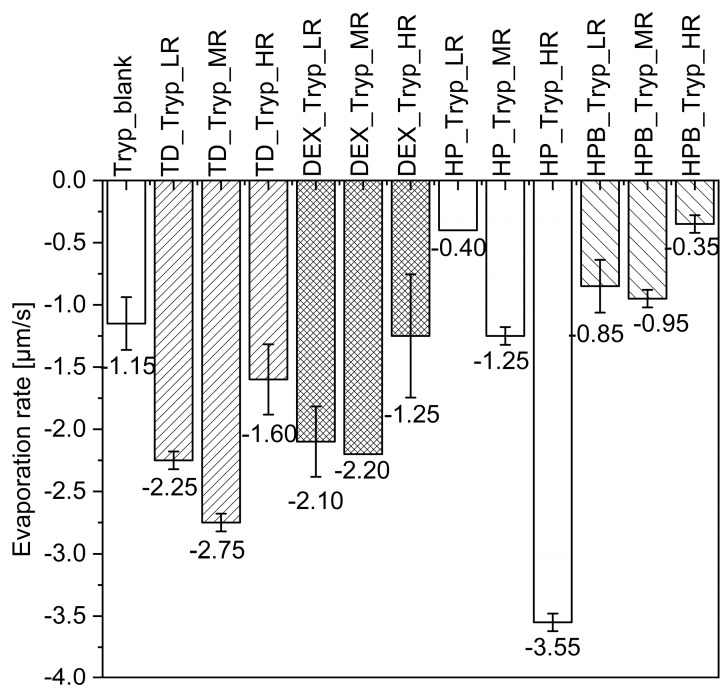
Evaporation rates of trypsin-saccharide droplets after MD for 15 min are presented. The blank corresponds to trypsin in water without saccharides. The measurements were performed in duplicates, and the bars show the mean value of the evaporation rates ± range (*n* = 3).

The previously reported, in-house-developed MD approach was applied [16]. Figure 4 summarizes the shapes of the dried droplets containing trypsin alone (a) or trypsin and one saccharide (b–n). Some of the dried trypsin particles, namely TD_Tryp_LR (b), HP_Tryp_LR (e), and HP_Tryp_HR (g), became very thin and bent and hence were not analyzable. Figure 4 shows that as TD is added to the trypsin solutions, more pancake-like particles are observed. The trypsin droplet in Figure 4a showed a thin and irregular shape after 15 min of drying. Overall, none of the dried trypsin droplets showed the typical “hat shape” morphology observed for catalase [30]. Figure 5 shows the evaporation rates of the droplets containing trypsin alone or with saccharides. All formulations with TD and DEX showed higher evaporation rates than the blank (−1.15 µm/s) at all ratios. HPB showed lower evaporation rates than the blank at all ratios (−0.35 to −0.85 µm/s), while HP showed higher evaporation rates than the blank at MR and HR. Increasing HP from LR (−0.40 µm/s) to HR (−3.55 µm/s) gave higher evaporation rates.

For all the other saccharides, the evaporation rate increased from LR to MR and decreased at HR. Using TD, DEX, and HPB at HR resulted in the lowest evaporation rates compared to when MR and LR saccharide ratios were applied, namely −1.60 µm/s for TD_Tryp_HR, −1.25 µm/s for DEX_Tryp_HR, and −0.35 µm/s for HPB_Tryp_HR. In summary, it can be said that for these three saccharides, the evaporation rates decrease as the amount of saccharide is increased to HR. The overall highest and lowest evaporation rates, which differ by a factor of ~10, were observed for the two cyclodextrins HP at HR (−3.55 µm/s) and HPB at HR (−0.35 µm/s). The observation of overall flatter trypsin droplets resulted in a smaller AR (Figure S3). The AR showed intermediate values between the highest and lowest values. The highest AR of dried trypsin droplets was detected with HPB at high concentrations, resulting in one of the largest droplets with a spherical shape (Figure 1j).

#### 3.2.2. Trypsin Enzymatic Activity during MD 

**Figure 6:**
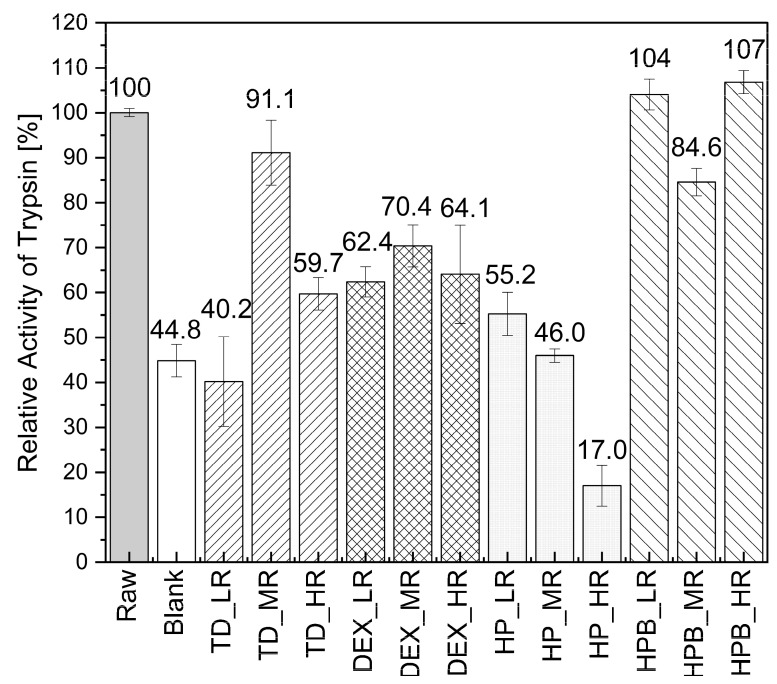
Relative activities of trypsin-saccharide droplets after MD for 15 min. The blank is trypsin in water, dried without saccharides. The reference Raw is the undried raw material. The bars present the mean relative activities ± error evaluated from dried droplets (doublets), analyzed in triplicate (*n* = 6).

Figure 6 shows the relative activities of dried trypsin droplets with and without different saccharides. Trypsin activities between 17.0% and 107% were observed, of which the highest was found with HPB at HR (107%) and HPB at LR (104%), being much higher than the activity of the blank (44.8%). The blank lost more than 50% activity compared to the two formulations and to the raw material (Raw), which presents the undried trypsin in solution (Figure 6). Of all formulations, only two resulted in lower trypsin activities during MD than the blank, namely TD at LR (40.2%) and HP at HR (17.0%).

All other ratios of TD and HP, as well as DEX and HPB applied in their specific concentrations, were shown to improve the relative activity of trypsin. HPB seemed to work the best in keeping trypsin active during drying, resulting in relative trypsin activities between 84.6% (at MR) and 107% (at HR) for all three different saccharide ratios applied. In relation to HPB, the higher substituted HP was not as successful in stabilizing trypsin during MD, resulting in rather low relative trypsin activities between 17.0% (at HR) and 55.2% (at LR). When trypsin was dried in the presence of DEX, the relative activities observed were between 62.4% (at LR) and 70.4% (at MR). Therefore, compared to trypsin dried without the addition of saccharides, DEX managed to preserve the activity of trypsin during MD. Especially using DEX at MR resulted in the highest trypsin activity compared to the other DEX ratios. Using the smallest saccharide TD in the formulations at MR resulted in higher activities of 91.1% than using it at HR, which led to a relative activity of 59.7%. For the formulations containing TD or DEX, the highest activities were obtained at MR, while with HP and HPB, a different behavior was observed. Namely, the activity either decreased with increasing content (for HP) or dropped only when applied at MR (for HPB), when compared to HPB at LR and HR, which both resulted in higher activities (Figure 6).

#### 3.2.3. Formation of Trypsin Particles and Activity during SD

The results of the Karl-Fischer titration of the spray-dried powders are as follows: the highest water content of 6.84 ± 0.20% was observed for trypsin SD without saccharides; when saccharides such as HP or HPB were present, much lower water contents of 4.07 ± 0.07% (for HP at HR) and 4.44 ± 0.48% (for HPB at HR) were observed. Hence, of the three powders, the higher substituted HP applied at HR led to the lowest moisture content, corresponding to a reduction of ~40% compared to trypsin without saccharides. After SD, no significant loss of trypsin activity was observed for any formulation (Figure S4B), all showing adequate activity values between 97.4 and 101%. The fact that the activities stay the same under all conditions during SD might indicate that trypsin remains active through protection by HPB and HP. However, of those two, only HPB could protect trypsin during both MD and SD (Figure S4A,B).

### 3.3. Modeling with Trypsin and HP or HPB

#### 3.3.1. Protein-Protein and Protein-Cyclodextrin Docking with Trypsin

To explain the different activities of trypsin in the MD experiments (Figure 6), a series of computer simulations were conducted on the molecular level, as shown in Supplementary Figure S5. CDs and their derivatives are widely used to improve the stability of proteins. Commercially available HPβCD is a mixture of a wide range of substitutions [31]. Two model substitutions are considered here. First, a β-cyclodextrin with four 2-hydroxypropyl groups linked to the O6 atom of glucose units 1, 3, 5, and 7 (Tet6-HPβCD, referred to as ‘HPB-LIKE’). Second, a β-cyclodextrin with seven 2-hydroxypropyl groups linked to the O6 atom of each glucose unit (Hep6-HPβCD, referred to as ‘HP-LIKE’). The two model systems test the dependency of the trypsin-cyclodextrin interactions in solution concerning their different levels of hydroxypropyl substitution. The six lowest free-energy non-overlapping docking structures between trypsin (red) and HPB-LIKE are shown in Supplementary Figure S6. The docking showed that both types should be able to inhibit protein-protein aggregation at high concentrations.

#### 3.3.2. Molecular Dynamics of Trypsin-Trypsin and Trypsin-Cyclodextrin Interactions

To also account for conformational flexibility, temperature, and concentration effects, molecular dynamics simulations of trypsin without and with the two different CDs were performed. The starting configuration included eight trypsin proteins on an equidistance cubic grid simulated for 500 ns at 63 °C. For the simulations with the CDs, each trypsin was surrounded by nine CDs, which led to a total number of 72 CD molecules. Thus, the simulations can also account for possible multimeric trypsin-trypsin, trypsin-CD, and CD-CD interactions in the aggregation process. Supplementary Figure S7A shows the structures at the end of the molecular dynamic simulations. In summary, the shift of the first two peaks to higher distances corresponds to the size of an HP-LIKE molecule (between 1.0 and 1.7 nm), which indicates that one CD is located between most protein dimers (Supplementary Figure S7B). Compared to the third peak of trypsin, the third peak with HP-LIKE is shifted by about 1.5–2 CDs. Because it only shifts the peaks but does not significantly lower the height of the peaks in the radial distribution function, the presence of HP-LIKE does not prevent aggregation (Figure S7(Ac)).

## 4. Discussion

### 4.1. Drying of Catalase and Trypsin without the Addition of Saccharides

During MD, as drying proceeds, the evaporation at the droplet surface increases, and at the same time, the concentration of the solids contained in the droplet rises compared to the liquids. For catalase, a hat shape was observed, while trypsin led to the appearance of overall flatter droplets. Tarasevich et al. discovered the influence of an increase in particle volume fraction during droplet drying on the resulting droplet shape [32]. If a droplet contains colloidal particles in a sufficiently large volume fraction, a pinned contact line is observed during drying as the particles accumulate. Furthermore, the authors mention that the volume fraction of the particles will increase at the center of the droplet [32]. This is one explanation for the hat shape of the catalase droplet without saccharides observed in our study. In contrast, a flat droplet (comparable to a pancake shape) would result from an average volume fraction inside the drying droplet [32]. In our study, this could explain the rather flat shape of trypsin droplets when dried without saccharides. According to Anton Paar, as a rule of thumb, proteins of larger size show higher viscosities [33]. Hence, as larger proteins are known to be more viscous, the larger catalase would be more viscous than the smaller trypsin, pinning the droplet rim and providing another explanation for the hat shape of the dried catalase droplet. During the droplet drying of trypsin alone, 50% activity loss was observed compared to the raw material. Furthermore, trypsin has a lower melting temperature of 50 °C [34,35] than catalase at approximately 58 °C [36]. This supports the observation that trypsin is more unstable than catalase. Catalase dried alone did not lead to a significant activity loss, supporting its higher activity than trypsin. The highest water content was observed for trypsin and catalase SD samples without saccharides. Interestingly, it has been observed that catalase is indeed stable when SD without saccharides is applied, as the loss in activity was much smaller during SD than during MD. For trypsin, adding saccharides to preserve its activity was indeed beneficial during MD, but no activity loss was observed during SD of trypsin without saccharides. During the SD of trypsin, the addition of saccharides was not beneficial. A reason could be that the drying process was faster during SD than MD. In a study by Haj-Ahmad et al., the decreased melting temperature of lysozyme (14.3 kDa) from 75.59 °C (SD lysozyme without excipients) to 72.60 °C (SD with β-CD) indicates destabilization of this enzyme by β-CD [37]. However, adding saccharides might be important as a bulk agent, an additive for particle engineering, or for conserving enzyme activity during storage.

### 4.2. Drying of Catalase and Trypsin in the Presence of TD

The catalase activity significantly decreased when dried with TD at MR, but increased with TD at HR. The evaporation rate decreased because water also interacts with saccharide molecules via hydrogen bonding. Thus, the transition to free water molecules, which evaporate, is rate-limiting. For both enzymes with TD, as the solid content increased from LR to MR, the evaporation rate increased as expected; however, at HR, the evaporation rate slowed. An explanation might be that sessile droplets with a higher viscosity are more prone to a decrease in the evaporation rate, as reported by Zhang et al. [38]. The hat shape is lost, and more pancake-like particles are observed when more TD is added to catalase. In turn, for trypsin, no hat shape was observed. As seen without saccharides, we address the loss of shape due to the increased solid content (leading to increased viscosity) of the formulations. This limits the solute diffusion and forms a pinned line of solutes [30] that leads to more oblong, thick particles looking like a pancake. For the larger catalase, a higher amount of TD preserved its activity better. For the smaller trypsin, TD at LR and HR could not protect from activity loss, but optimum protection was found at MR. Using disaccharides and a trisaccharide, Giuffrida et al. [39] have demonstrated that in amorphous matrices, an optimum S/P ratio exists, a balance between protein and saccharide exists, and perturbations to the protein structure are minimal. Based on these distinct results, it was decided to use SD catalase with TD at MR and HR to understand how the results from MD would translate to lab-scale SD. The addition of TD at HR led to a lower moisture content. Different from MD, in SD, higher activities were observed when SD was in the presence of TD at MR and HR. A potential explanation for these observations might be the capacity of different processes (i.e., MD and SD) to remove water from the system. We can assume that the heat-mass exchange during MD is less efficient than in SD, and water is not so well removed from the system. Thus, due to the higher molecular mobility of the system, more perturbations to the protein structure might exist [40]. Hence, depending on the drying process, different S/P ratio optimums might exist and should be considered [41].

### 4.3. Drying of Catalase and Trypsin in the Presence of DEX

During MD, it was observed that the addition of DEX showed lower evaporation rates for higher droplet shapes. The loss of the hat shape for dried droplets of some formulations containing higher saccharide content can be explained by the increase in solid concentration during the evaporation of water during drying. Hence, the drying droplet becomes more viscous and, thereby, prevents the diffusion of the solids [42]. In this way, the hat shape is lost, as the saccharides cannot move towards the droplet surface anymore. DEX attracts attention here as it is the only one for which the evaporation rate decreases as its amount increases in the catalase formulations. Most probably, the water could not evaporate very well due to the high viscosity of the formulations (observed during the preparation and handling of these formulations), reducing the water diffusion. Hence, higher DEX concentrations lead to more water being retained, thereby reducing the evaporation rate [43]. It might be that the large size of DEX leads to larger droplets with a larger surface area. In general, larger saccharides such as DEX are limited in their translation diffusivity. Therefore, they can only diffuse slowly from the droplet surface toward the droplet center. The formation of a crust happens much earlier than with the other saccharides, leading to larger particles [44]. Adding DEX at HR to the trypsin formulation resulted in higher trypsin activities than if DEX was used at MR or LR (Figure 6). This observation could be explained by DEX’s ability to preserve trypsin’s activity by limiting its movement. This steric hindrance and the vitrification theory based on its high glass transition temperature could be possible explanations [45].

### 4.4. Drying of Catalase and Trypsin in the Presence of HP and HPB

During the MD of trypsin, it was observed that the more saccharides are present, the higher the dried droplets will get. However, the higher substituted HP did not follow this trend and showed flatter droplets for all ratios. Nonetheless, the obtained evaporation rates align with the observed shapes, assigning higher shapes to slower evaporation, except for HP. Adding the lower-substituted HPB during drying had a beneficial effect on trypsin’s activity. The addition of HP-LIKE led to a higher binding affinity for trypsin. The higher the HP or HPB content, the flatter the shape. As the solid content increases, the viscosity increases, and consequently, the Marangoni flow is reduced. HP showed comparable evaporation rates with both enzymes during MD, resulting in higher evaporation rates with more HP applied. There might be a significant connection between activity and evaporation rate [19], because higher evaporation rates cause faster drying and lead to activity loss. During MD, it was observed that the CDs can protect the protein either by binding to its hydrophobic parts or acting on its surface [41,46]. These effects strongly depend on the protein type and the CDs’ degree of substitution. Even though the exact mechanism of how the degree of substitution influences protein stabilization is unclear, small changes can already have an effect [41]. For catalase, HP was slightly superior at LR but comparable to HPB at HR. Due to its large size, it is rather difficult for catalase to approach the hydrophobic pocket of CDs [47,48]. Efstratiou et al. reported similar observations during the sessile droplet drying of fetal bovine serum [49]. The authors hypothesize that if evaporation rates are increased, more water will evaporate, resulting in the loss of stabilizing hydrogen bonds [49]. However, when saccharides are added to the protein, the obtained particles are smooth and spherical [13,50]. In summary, the MD is less efficient and slower than the SD. Due to these interesting and distinct results between the different CDs, they were chosen for SD to understand how the results observed during the MD would translate to SD. We observed that adding HP or HPB to trypsin during SD reduced the moisture content, resulting in the lowest one with HP. It was observed that trypsin activity was much more affected during MD than during SD, resulting in very low activity with HP. However, it was shown that during MD, HPB was significantly more successful in preserving trypsin’s activity than HP. The observations during SD aligned well with those during MD, namely that the highest trypsin activities were obtained with HPB at LR or HR. For a better understanding of the CDs’ mechanism, we carried out molecular modeling experiments with trypsin, HP-LIKE, and HPB-LIKE molecules.

### 4.5. Molecular Modeling of Trypsin and Cyclodextrin Systems

The docking results show that HP-LIKE has a higher affinity for trypsin than HPB-LIKE. Protein-CD-docking demonstrated the formation of strong bonds between trypsin and the HPB-LIKE CD (Supplementary Figure S6a). At sufficiently high concentrations, HPB can prevent this aggregation in bulk solutions. Still, at air-water interfaces, it is less effective due to its tendency to concentrate at these interfaces. The molecular dynamics simulations between trypsin molecules showed the formation of compact multimers that might form trypsin aggregates. It was mentioned that this trypsin-trypsin aggregation could be inhibited by the addition of HPB-LIKE, for which the aggregation of trypsin was low. This presents one possible stabilization mechanism of HPB-LIKE on trypsin. HP-LIKE, on the other hand, does not only bind to trypsin molecules but also tends to self-assemble like a “glue”. Hence, as it does not prevent trypsin from aggregation, these findings present possible destabilization mechanisms of HP-LIKE on trypsin. These outcomes have been confirmed by observations from the drying experiments in this study, in which a stabilizing effect was seen when HPB was present during the drying of trypsin. A reason for the different observations during MD and SD might be the higher temperature during MD, which seemed more critical than trypsin being present at the interface, and the addition or absence of some excipients. Although a higher air-liquid interface is present during SD, the higher activities might result from improved evaporation and cooling. As the HP-LIKE contains three HP-groups more (larger) than the HPB-LIKE, steric hindrance between the HP molecules might be at work [47,48,51]. As Schönbeck et al. reported, this might prevent trypsin from interacting with the saccharide, resulting in self-aggregation with other trypsin molecules [51]. The outcome of molecular modeling has set a strong precedent for further experimental characterization of molecular interactions among protein, saccharide, and water using infrared, Raman, or nuclear magnetic resonance spectroscopy, especially during the drying process. In addition, speciation and quantitation of the formed aggregates using chromatographic or spectroscopic techniques will help corroborate the findings of the computational study and relate to the observed in-process activity of the dried particles.

## 5. Conclusions

This study aimed to reveal whether different saccharides in different amounts could preserve the activity of trypsin and catalase during MD and/or SD. Our results show that during MD, the smallest saccharide TD seems to preserve the activity of the larger catalase the best. Using TD at MR and HR during MD and SD showed that it is more successful in stabilizing catalase during SD. For the smaller enzyme trypsin, the presence of the saccharide HPB during MD was beneficial. We observed that, of the different CDs, HPB preserved trypsin’s activity well at all ratios. However, HP successfully preserved trypsin’s activity during SD only, whereas HPB kept trypsin active during both MD and SD. The SD results showed that the MD approach could predict some of the trends observed during SD, even though the two examined drying approaches markedly differed in their dimension, configuration, scale, and speed. Therefore, using trypsin and catalase, once again, the MD approach proved to be a valuable screening technique for different formulations regarding their activity preservation capabilities before going into SD. Basically, the MD represents a “worst-case” scenario that could help to exclude specific formulations, because formulations that did not give adequate results in MD will not give adequate results in SD either. Hence, MD can serve as a prior screening tool to select SD formulations for industrial applications. The different observations with CDs during trypsin drying but not during catalase drying might suggest a dependence between size and the amount of TD needed in the case of catalase. As catalase is larger than trypsin, nearly by a factor of ~10, more TD might be required to protect this larger enzyme during drying. This confirms that the stabilization by ß-CDs during drying strongly depends on the nature of the enzymes. Molecular docking and dynamics simulations helped to understand the important interactions underlying the stabilization mechanism of trypsin by CDs. HP’s tendency to move to the hydrophobic air-water interface leads to trypsin molecules being carried away from the center, followed by activity loss. This is true for MD but not applicable to the SD of trypsin, which did not show significant activity loss. In this study, molecular modeling results showed that the higher substituted HP inhibits a higher affinity towards trypsin and itself, presenting a higher aggregation propensity. As discussed, SD results align well with these observations, but MD results do not. Future studies of a variety of proteins and excipients would help to predict the activity of biotherapeutics.

## Figures and Tables

**Table 1:** Sugar-to-Protein (S/P) ratios, solid contents, and composition of trypsin or catalase formulations **for MD**, with or without the saccharides TD, DEX, HP, or HPB in water. MD = miniaturized drying, LR = low ratio, MR = medium ratio; HR = high ratio.

Composition of Formulations	Saccharide Used	Saccharide Concentration [mM]	S/P Ratio	Solid Content [wt.%]
Tryp_blank	-	-	-	9.9
TD_Tryp_HR	TD	400	95.2	25.1
TD_Tryp_MR	TD	200	47.6	17.6
TD_Tryp_LR	TD	50	11.9	11.9
HP_Tryp_HR	HP	75.6	18.0	25.0
HP_Tryp_MR	HP	37.8	9.00	17.5
HP_Tryp_LR	HP	1.5	0.36	13.0
HPB_Tryp_HR	HPB	75.6	18.0	21.2
HPB_Tryp_MR	HPB	37.8	9.00	15.6
HPB_Tryp_LR	HPB	1.5	0.36	10.2
DEX_Tryp_HR	DEX	3.75	0.89	20.6
DEX_Tryp_MR	DEX	1.85	0.44	15.3
DEX_Tryp_LR	DEX	0.75	0.18	10.2
**Cat_blank**	-	-	-	10.1
TD_Cat_HR	TD	400	1000	25.3
TD_Cat_MR	TD	200	500	17.6
TD_Cat_LR	TD	50	125	12.1
HP_Cat_HR	HP	75.6	189	25.0
HP_Cat_MR	HP	37.8	94.5	17.5
HP_Cat_LR	HP	1.5	3.75	12.9
HPB_Cat_HR	HPB	75.6	189	17.2
HPB _Cat_MR	HPB	37.8	94.5	14.9
HPB _Cat_LR	HPB	1.5	3.75	10.2
DEX_Cat_HR	DEX	3.75	9.38	20.5
DEX_ Cat_MR	DEX	1.85	4.63	15.2
DEX_Cat_LR	DEX	0.75	1.88	10.1

## Data Availability

The data presented in this study are available on request from the corresponding author. The data are not publicly available due to company data policy.

## Supplementary Materials

The following supporting information can be downloaded at: https://www.mdpi.com/article/10.3390/pharmaceutics15102504/s1, Figure S1: AR of catalase-saccharide droplets after MD for 15 min is presented; Figure S2: Relative activities of catalase—TD (A) droplets after MD and (B) powders after SD are shown; Figure S3: AR of trypsin-saccharide droplets after MD for 15 min is presented; Figure S4: Relative activities of trypsin—HP/HPB (A) droplets after MD and (B) powders after SD are shown; Figure S5: Lowest-energy dimers of trypsin from protein-protein docking. The first trypsin chain in the cartoon representation is depicted in red, and the second chain is blue; Figure S6: Lowest free-energy non-overlapping docking structures between trypsin (red) and (A) HPB-LIKE (van der Waals spheres) or (B) HP-LIKE (van der Waals spheres); Figure S7: (A) Structures after 500 ns of molecular dynamics simulations in an aqueous solution of (a) eight trypsin molecules (8 mM) at 63°C (b) eight trypsin molecules (8 mM) + 72 HPB-LIKE (79.2 mM) (c) eight trypsin molecules (8mM) + 72 HP-LIKE (64.8 mM). (B) Radial distribution functions between the centers of mass of the proteins during the last 400 ns of the molecular dynamic’s simulations of eight trypsin molecules (red line), eight trypsin molecules + 72 HPB-LIKE (blue line), as well as eight trypsin molecules + 72 HP-LIKE (purple line); Figure S8: (A) Final structures after 500 ns of molecular dynamics simulations of a layer of water between two air-water interfaces of (a) eight trypsin molecules (8 mM) at 63 °C (b) eight trypsin molecules (8 mM) + 72 HPB-LIKE (79.2 mM) (c) eight trypsin molecules (8 mM) + 72 HP-LIKE (64.8mM). The aqueous phase is shown in blueish gray, while the gas phase is white. The interface area is highlighted by the wave-like surfaces on the aqueous phase. (B) Radial distribution functions between the centres of mass of the proteins during the last 400 ns of the molecular dynamic’s simulations at air-water interfaces of eight trypsin molecules (red line), eight trypsin molecules + 72 HPB-LIKE (blue line), as well as eight trypsin molecules + 72 HP-LIKE (purple line). References [52,53,54,55,56,57,58] are cited in the supplementary materials.
