# Supplementary material for: Investigation of the Impact of Saccharides on the Relative Activity of Trypsin and Catalase after Droplet and Spray Drying
Source: Pharmaceutics. 2023 Oct 21;15(10):2504. doi: 10.3390/pharmaceutics15102504 (PMC10609839; doi:10.3390/pharmaceutics15102504)
Supplement: Supplementary file 1 [file pharmaceutics-15-02504-s001.zip › pharmaceutics-2662620-supplementary.pdf]

pharmaceutics

MDPI

Supplementary Material: Investigation of the impact of saccharides on the relative activity of trypsin and catalase after droplet and spray drying

Johanna Dieplinger; Christina Moser; Gerhard König; Joana T. Pinto; Amrit Paudel

1. Materials and Methods

1.1. Molecular Docking and Molecular Dynamics Simulations

To identify the lowest-energy non-overlapping binding positions of the two kinds of cyclodextrin to trypsin, molecular docking was performed with the Dock module of MOE. The receptor atoms were held fixed throughout the course of the simulations. Initial placement of the cyclodextrins was performed using the Triangle Matcher method, and these positions were scored using the London dG approach. The top 100 poses were then energy minimized and the binding free energy of the cyclodextrin was estimated using the GBVI/WSA dG function.

To create simulation boxes with air-water interfaces, the box length in the z-direction was doubled, and constant volume simulations were performed. It shall be noted that the evaporation of water molecules was neglected for performing these simulations [1]. Langevin dynamics were performed with a time step of 2 fs using SHAKE constraints on all hydrogens[2], a friction coefficient of 1 ps^{-1}, and a temperature of 336.15 K. The constant pressure simulations maintained the pressure at 1 atm with a Monte Carlo barostat[3]. The cut-off was 12 Å using force-switching[4] with a switching region of 2 Å. Electrostatic interactions were treated with the Particle Mesh Ewald method[5]. After 5000 steps of energy minimization with the steepest descent, the structure was heated and equilibrated for 0.125 ns, followed by 500 ns of production. The trajectories were processed with cpptraj[6] to calculate the radial distribution functions of the centers of mass of all proteins.

2. Results

2.1. AR of catalase

Figure S1 presents the AR observed during MD of the different formulations containing catalase alone or with saccharides. The overall lowest AR was observed for DEX at LR (0.07) and MR (0.07), whereas the overall highest AR was observed for HPB at LR (0.35). However, the AR of HP at LR (0.33) and the blank (0.34) are similar. All the formulations containing catalase and TD, HP, or HPB showed a decrease in AR by a factor of 3.5, 2.5, and 1.9, respectively, as the saccharide contents were increased from LR to HR. DEX, as the only exception, showed an increase in AR by a factor of 2.5, as its content was increased from LR to HR.

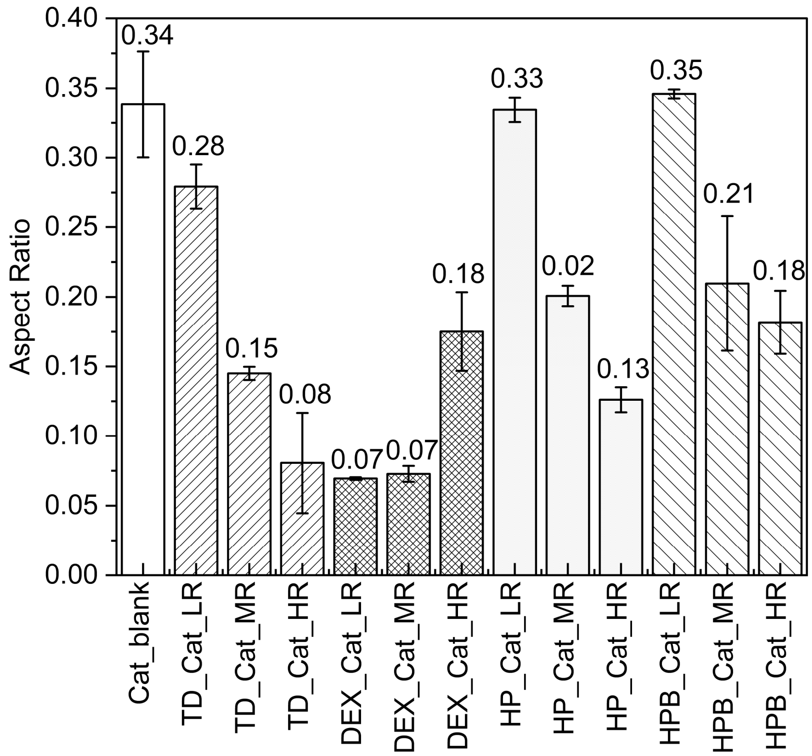

Figure S1. AR of catalase-saccharide droplets after MD for 15 min are presented. The blank corresponds to trypsin in water dried without saccharides. The bars present the mean value of AR ± range (n=2).

Figure 1k shows the shape of DEX at LR with the lowest AR of 0.07, representing the flattest of all. In contrast, Figures 1a, b, e, and h clearly show that the largest AR of 0.28 – 0.35 (Figure 1) can be associated with the typical "hat shape" morphology. Furthermore, except HPB, trends in the increase of drying rate have been observed with the increasing content of all other saccharides. Interestingly, the dried droplets with the highest TD and lower DEX concentrations showed the flattest droplet shapes. Compared to the droplet shapes summarized in Figure 1 d) and 1 k), their morphology became more oblong and the typical "hat-shape" was lost as the saccharide content was increased.

Catalase in-process stability during MD vs. SD

Figure S2 summarizes a paired comparison plot of the obtained relative activity values for the catalase samples after MD (Figure S2A) and SD (Figure S2B), with and without saccharides. When comparing TD at MR and HR, significantly higher activity is observed for TD at HR (Figure S2A). However, catalase dried without saccharides seems significantly better.

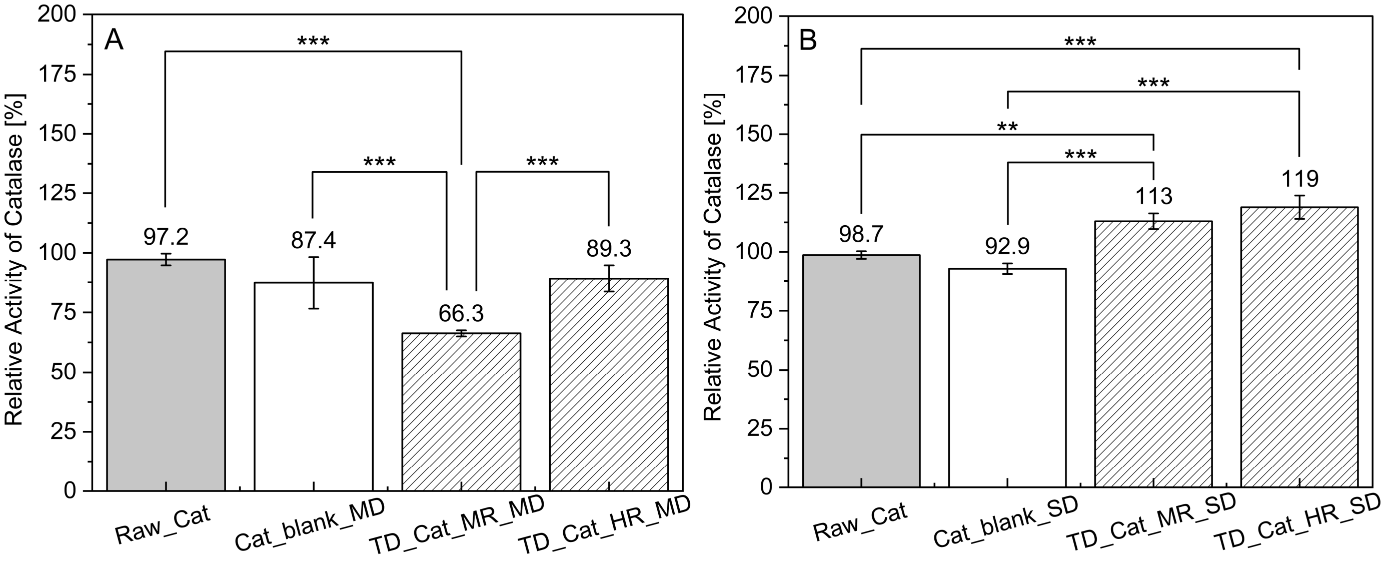

Figure S2. Relative activities of catalase – TD **(A)** droplets after MD and **(B)** powders after SD are shown. The blank corresponds to catalase in water dried without saccharides and the reference Raw_Cat to catalase raw material before drying. (** = Significance at p<= 0.01; *** = Significance at p <= 0.001). The bars present the mean of the relative activities ± standard deviation (n=3). Bonferroni and paired comparison were used.

As shown in Figure S2B for SD samples, a significant increase ($p \leq 0.001$) of catalase activity for the formulations containing TD was observed, indicating that addition of TD to catalase is beneficial to the enzyme activity during SD. However, based on Figure S2B it is evident that catalase spray dried without any saccharides seems to be quite stable when dried at the chosen SD conditions. Furthermore, when spray drying catalase in the presence of TD, a higher relative activity of 119% was observed. No matter, if TD was used at HR or MR, the relative activities of catalase, were consistently above 100%, higher than the blank. This means, that the higher content of TD was better at protecting the enzyme.

2.2. AR of Trypsin

Figure S3 presents the dried droplet's aspect ratios (AR) of the different formulations. The lowest AR was observed for TD at MR (0.03) while the highest was observed for HPB at HR (0.21). In the presence of TD, the AR increased by a factor of ~3.33 from MR to HR. In the presence of DEX, the AR increased by a factor of ~1.90 from MR (0.08) to HR (0.15), and for HPB, the AR increased by a factor of 1.50 from MR (0.14) to HR (0.21). The AR of HP at MR showed the same value as AR for TD at HR. Due to the mentioned droplet bending after 15 min of drying, only one droplet of the blank (0.16) and of DEX at LR (0.07) could be analyzed according to AR (Figure S3). At lower contents, DEX decreased the AR but increased the AR when used in higher concentrations. When the content of HP and HPB was increased, the droplet volume, as well as the AR, increased. Because of this opposite behavior of the two saccharides during MD, they were chosen for the following SD experiments.

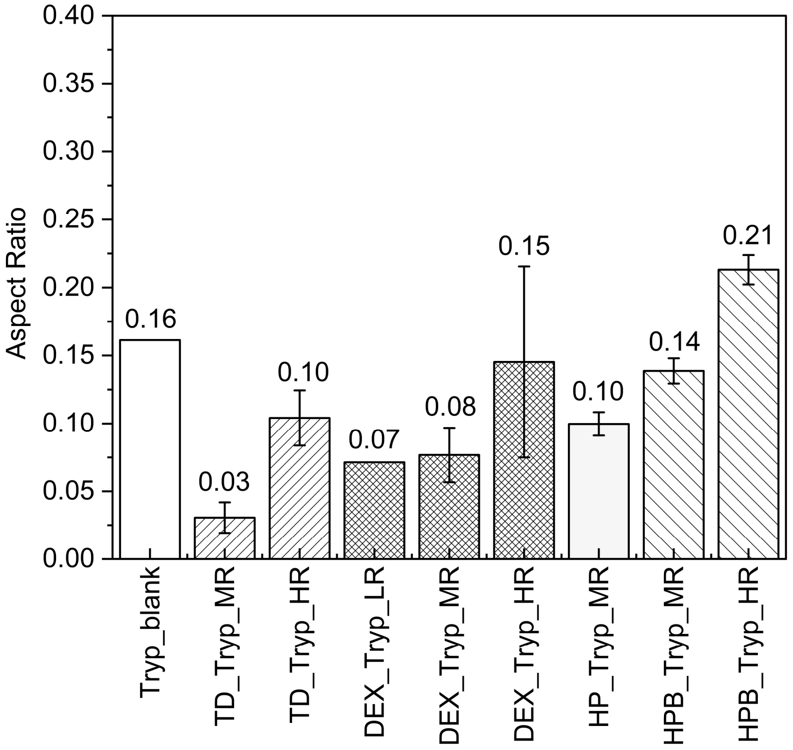

Figure S3. AR of trypsin-saccharide droplets after MD for 15 min are presented. The blank corresponds to trypsin in water dried without saccharides. The bars present the mean value of the AR ± range (n=2). NOTE: The missing values for HP and HPB are due to the droplet bending of these droplets during drying.

2.4. Trypsin in-process stability during MD vs. SD

Figure S4 compares the specific formulations chosen after analysis by MD and following SD experiments.

Figure S4B presents the activities of the different spray-dried trypsin powders with and without the chosen saccharides HP and HPB at HR. The activity of the undried sample (Raw_Tryp) and trypsin after spray drying without saccharides was very similar. The trypsin activities of all formulations after SD were observed to be at 97.4% for HP at HR, 98.5% for HPB at HR, and 101% for the blank containing no saccharides (Figure S4B). The high trypsin activities observed for all formulations after SD suggest that there might be no significant benefit in adding saccharides like HP and HPB during SD. Moreover, unlike MD, no (partial) unfolding of trypsin is observed during SD. During MD, heating is applied without airflow, whereas during SD, heating is applied using airflow. Hence, during SD, as the solvent evaporates, a cooling effect is generated by the drying airflow, which protects the droplets from experiencing temperatures as high as the melting temperature of trypsin.

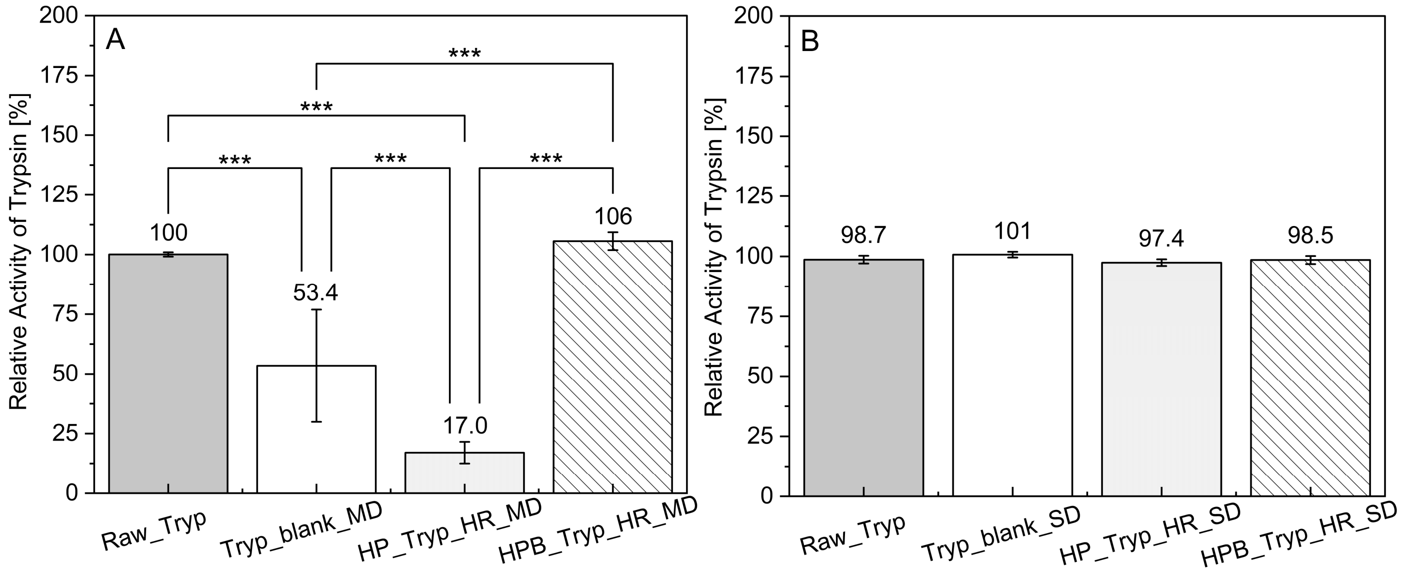

Figure S4. Relative activities of trypsin – HP/HPB **(A)** droplets after MD and **(B)** powders after SD are shown. The blank corresponds to trypsin in water without saccharides and the reference Raw_Tryp to trypsin raw material before drying. (*** = Significance at p <= 0.001). The bars present the mean of the relative activities ± standard deviation (n=3). Bonferroni and paired comparison were used.

2.5. Molecular Modeling with Trypsin and HP or HPB

2.5.1. Protein-Protein and Protein-Cyclodextrin Docking with Trypsin

The six lowest-energy trypsin-trypsin complexes are depicted in Figure S5. The most stable complex involves contacts between residues 27-33, 77-83, and 149-156 of the first protein chain (red) and residues 43-48, 101-103, 149-153, 197-198, and 218-219 of the second protein chain (blue). The binding of the second chain in modes b, c, and f exhibit partial overlap with mode a, which means that they are sterically mutually exclusive. Thus, no third protein can bind in modes b, c, or f, if there is already a protein bound in mode a. However, due to the multiple interaction possibilities in the same region, binding is entropically favored. This implies an increased tendency towards dimerization.

In addition to the binding of the second chain on the right side of the first chain in Figure S5, there are two binding modes at the bottom and on the left side that would allow the creation of cross-networks. Binding mode d attaches to the bottom of the first chain, while binding mode e interacts with the left side of the first chain. Since modes d and e are not overlapping, they can be formed simultaneously. Thus, there are three low-energy ways in which trypsin can aggregate.

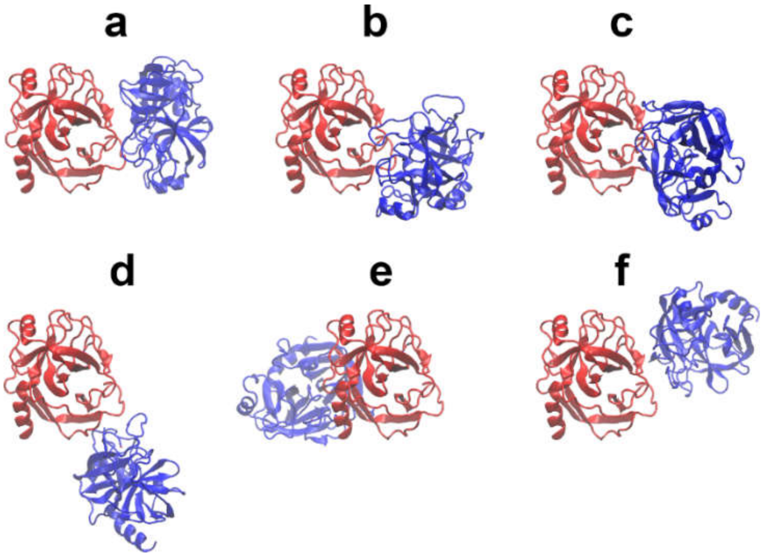

Figure S5. Lowest-energy dimers of trypsin from protein-protein docking. The first trypsin chain in the cartoon representation is depicted in red and the second chain in blue. The binding modes are ranked based on their interaction energies (E refine) from MOE of **a)** -197.1 kcal/mol **b)** -190.7 kcal/mol **c)** -170.8 kcal/mol **d)** -126.8 kcal/mol **e)** -111.7 kcal/mol **f)** -111.3 kcal/mol, where more negative values indicate stronger binding.

The docking results were further confirmed by two 500 ns molecular dynamics simulations of two trypsin molecules at different temperatures. In a simulation at 315.15K (42°C), almost exclusively complexes from the cluster of modes a, b, c, f on the right side of the first chain were observed, which are in line with the interaction energies from protein-protein docking. This implies that, at 42°C, trypsin will mainly form dimers. In a simulation at an elevated temperature of 336.15 K (63°C), mainly binding modes b, d, and e were observed. This indicates that in solution trypsin presents a higher aggregation tendency at elevated temperatures because the three binding modes allow the formation of crosslinks. Furthermore, it shall be noted, that partial unfolding is indeed another important cause for the aggregation of proteins [7].

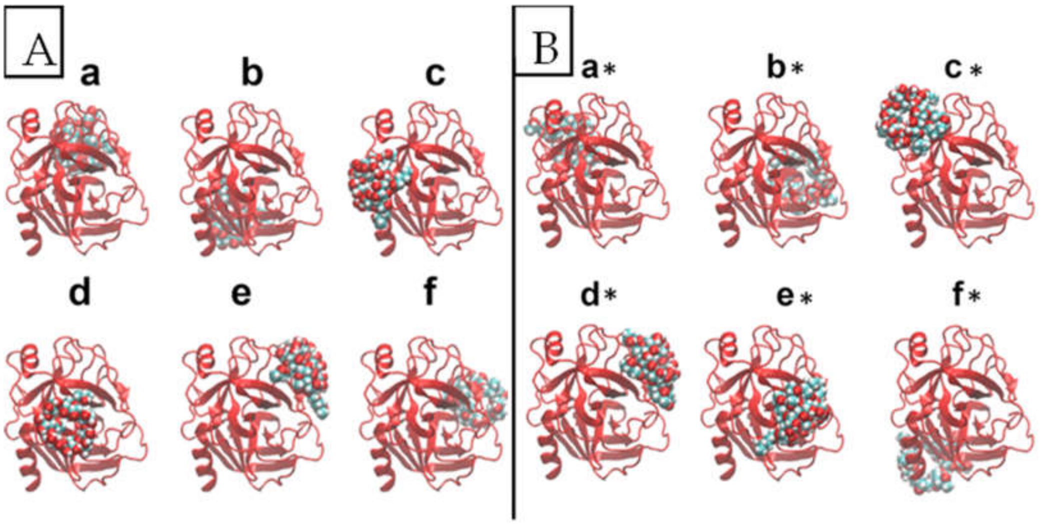

Figure S6. Lowest free-energy non-overlapping docking structures between trypsin (red) and **(A) HPB-LIKE** (van der Waals spheres) or **(B) HP-LIKE** (van der Waals spheres). The six binding modes are ranked based on their binding free energy estimates from molecular docking of **(A) a)** -10.4 kcal/mol **b)** -9.1 kcal/mol **c)** -8.9 kcal/mol **d)** -8.6 kcal/mol **e)** -8.5 kcal/mol **f)** -8.5 kcal/mol. Molecular docking with **(B)** gave **a*)** -11.5 kcal/mol **b*)** -10.7 kcal/mol **c*)** -9.8 kcal/mol **d*)** -9.0 kcal/mol **e*)** -9.0 kcal/mol **f*)** -9.0 kcal/mol. Note: More negative values indicate stronger binding.

The best binding conformation (Figure S6a) exhibits an estimated binding free energy of –10.4 kcal/mol, which indicates strong binding between trypsin and HPB-LIKE. Such selective binding to the folded state might stabilize the protein in solution in its native conformation. However, to prevent aggregation, the cyclodextrin should ideally attach to the protein interfaces that are involved in multimerization and block the protein-protein binding. A comparison between the cyclodextrin docking positions in Figure S6A and protein-protein binding structures in Figure S5 reveals that the strongest protein binding pose of HPB-LIKE can only interfere with protein-protein interaction f in Figure S5. HPB-LIKE binding position b in Figure S6A can block the protein-protein interaction e of Figure S5. The best docking pose to prevent aggregation is position f of Figure S6A, as it prevents protein-protein interactions a, b, c, and f of Figure S5. However, since the affinity of HPB-LIKE towards binding mode f is energetically less favourable than the others in Figure S5, this binding position will be only occupied once the lower-energy sites are saturated with HPB-LIKE. This indicates that the capability of HPB-LIKE to prevent aggregation is concentration-dependent. Compared to HPB-LIKE, the best binding pose of HP-LIKE exhibits a stronger binding affinity towards trypsin (–11.5 kcal/mol compared to –10.4 kcal/mol), which probably can be attributed to interactions of the three additional 2-hydroxypropyl groups. Also, the positions of the binding sites differ between the two forms of cyclodextrin. Binding mode a of HP-LIKE can only interfere with the protein-protein binding mode e of Figure S5. Binding mode d of HP-LIKE can block protein-protein interactions a, b, c, and f. Binding mode e of HP-LIKE occupies the protein-protein binding sites b and c, while binding mode f of HP-LIKE blocks the protein-protein binding sites e. For HP-LIKE, the six lowest free-energy non-overlapping docking structures to trypsin (red) are depicted in Figure S6B (a* - f*). Thus, also the capability of HPB-LIKE to prevent aggregation is expected to be concentration-dependent, as binding mode d will mainly be occupied once the binding sites of modes a, b, and c are occupied.

2.5.2. Molecular Dynamics of Trypsin-Trypsin and Trypsin-Cyclodextrin interactions

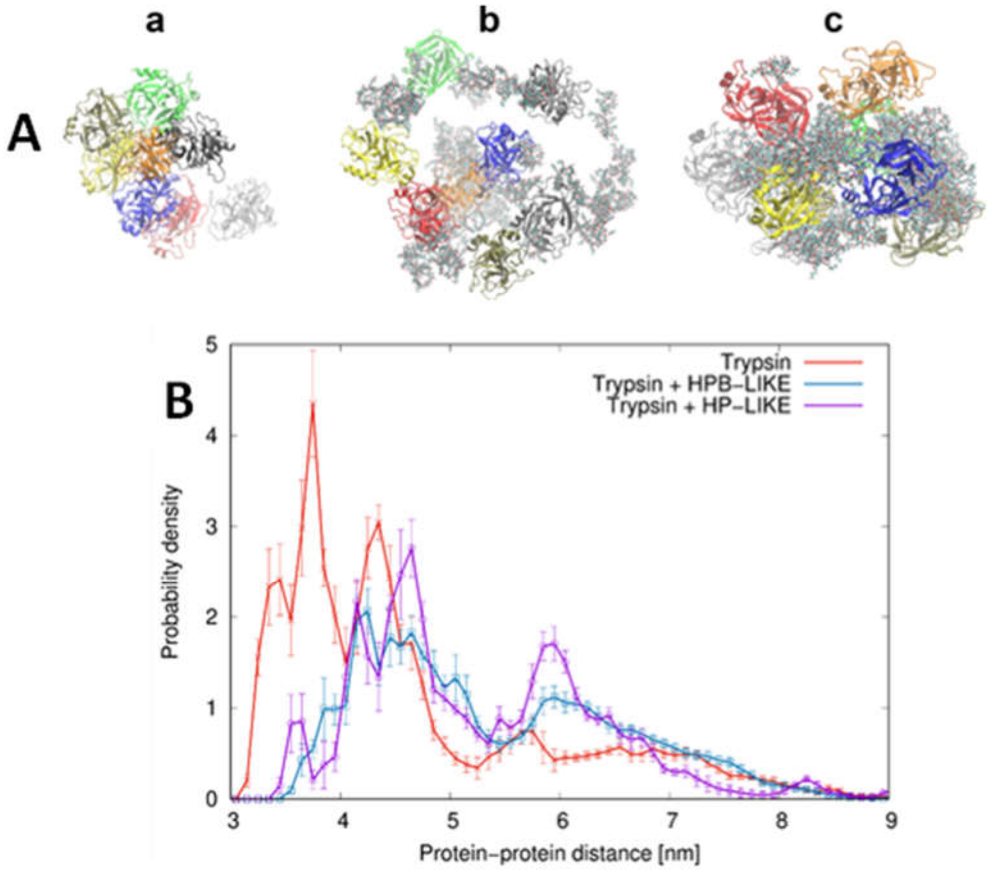

Figure S7. (A) Structures after 500 ns of molecular dynamics simulations in aqueous solution of **a)** 8 trypsin molecules (8mM) at 63°C **b)** 8 Trypsin molecules (8mM) + 72 HPB-LIKE (79.2mM) **c)** 8 Trypsin molecules (8mM) + 72 HP-LIKE (64.8mM). **(B)** Radial distribution functions between the centres of mass of the proteins during the last 400 ns of the molecular dynamic's simulations of 8 trypsin molecules (red line), 8 Trypsin molecules + 72 HPB-LIKE (blue line), as well as 8 Trypsin molecules + 72 HP-LIKE (purple line). Probability density peaks at short protein-protein distances indicate aggregation, while a flat distribution would indicate no protein-protein interactions. The mean values with standard errors from 7 blocks are presented.

Subplot a of Figure S7A demonstrates that an 8mM trypsin solution tends to form stable multimers, which is likely a pre-stage to forming larger aggregates. When 79.2mM of HPB-LIKE are added (Figure S7A b), the cyclodextrins bind between the trypsin molecules, which leads to a more chain-like structure without a compact trypsin multimer. This is an indicator that HPB-LIKE can inhibit protein-protein aggregation. When 64.8mM of HP-LIKE are added, they bind between the trypsin molecules, thus preventing direct protein-protein interactions. However, in contrast to HPB-LIKE, there is also a strong tendency for the cyclodextrins to bind to each other. Because of the strong interactions between both types of molecules, HP-LIKE acts as a glue that keeps the trypsin molecules together. Therefore, the simulations can explain the experimental findings in Figure S8A, where trypsin exhibits a lower activity in the presence of HP and increased activity in the presence of HPB.

To quantify protein-protein aggregation, radial distribution functions between the centres of mass of all eight trypsin proteins were calculated over the last 400 ns of the simulations (Figure S7B). The radial distribution functions report the probability of finding two proteins at a certain distance relative to a homogenous distribution according to the concentration of the proteins. A flat radial distribution function of one indicates a homogeneous distribution. Therefore, it would imply no interactions between the proteins, while peaks at short distances imply protein-protein interactions. For the simulation with eight trypsin molecules (red line in Figure S7B), three peaks can be seen at distances between 3 and 4.5 nm. The radius of gyration of trypsin is about 1.7 nm, which means that, on average, two trypsin molecules are in direct contact with each other at about 3.4 nm, corresponding to the first peak. However, trypsin is not perfectly spherical, so the first peak indicates an interaction along the shorter side of trypsin (like in protein-protein binding modes a and e of Figure 7), while the second peak indicates an interaction along the longer side of trypsin (like in protein-protein binding modes b, c, d, and f of Figure 7). The third peak at about 4.4 nm probably corresponds to a third protein attaching to the sides of an existing dimer (Figure S7A a). In contrast, the radial distribution function of trypsin with HPB-LIKE (blue line in Figure S7B) does not exhibit high probability peaks at short distances. This indicates that there is very little aggregation between the proteins. Two small peaks arise around a distance of 4.1-4.5 nm, corresponding to the radii of gyration of two trypsin molecules plus the average size of an HPB-LIKE (between 0.8 and 1.6nm). This corresponds to an alternating chain of trypsin molecules and cyclodextrins as observed in Figure S7A b. The radial distribution function of trypsin with HP-LIKE (purple line in Figure S7B) resembles the one observed for pure trypsin (red), but the peaks are shifted to longer distances and are slightly lower.

2.5.3. Molecular Dynamics Simulations at Air-Water Interfaces

Another factor that might promote protein aggregation is the presence of air-water interfaces, such as encountered on the surface of a droplet or by the formation of air bubbles. Since the gas phase cannot form hydrogen bonds, air-water interfaces represent a hydrophobic environment. Therefore, proteins with hydrophobic patches on the surface or other molecules might be found at increased concentrations at air-water interfaces. Supplementary Figures S8 A and S8 B summarize all findings of the molecular dynamics. Overall, the results indicate that HP-LIKE not only serves as a glue between trypsin

molecules but also has a solid propensity to attach the trypsin aggregates to the air-water interface, which might further destabilize the proteins. To test the effect of air-water interfaces on trypsin, 500 ns molecular dynamics simulation with a gas phase along the z-direction were performed. The structures resulting from the simulations with air-water interfaces are shown in Figure S8A.

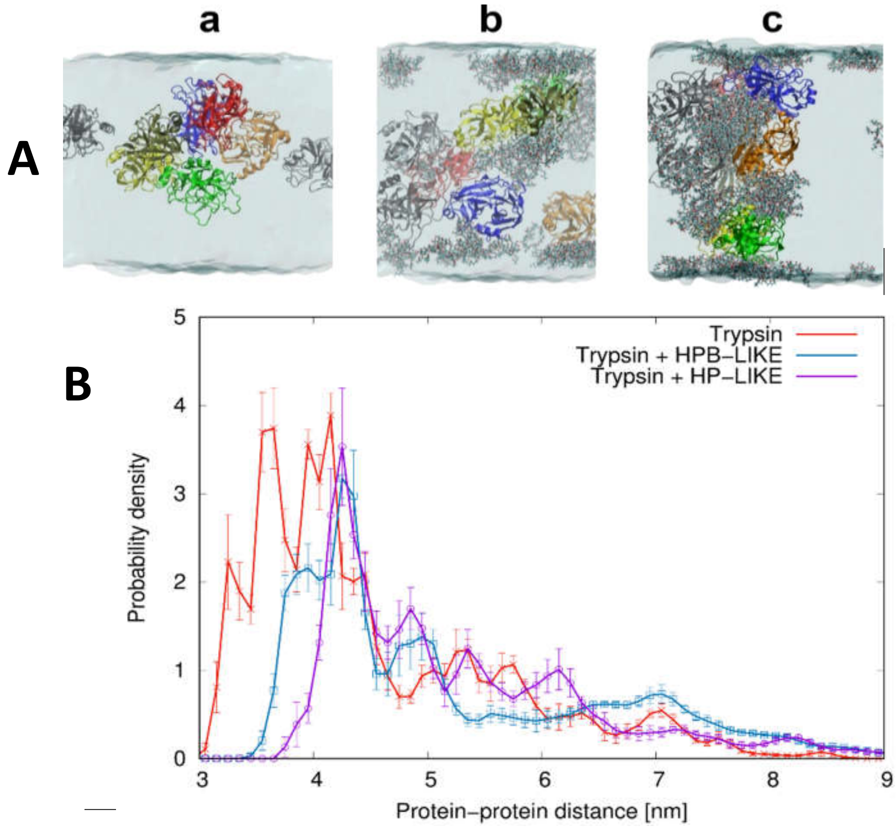

Figure S8. (A) Final structures after 500ns of molecular dynamics simulations of a layer of water between two air-water interfaces of **a)** 8 trypsin molecules (8mM) at 63°C **b)** 8 Trypsin molecules (8mM) + 72 HPB-LIKE (79.2mM) **c)** 8 Trypsin molecules (8mM) + 72 HP-LIKE (64.8mM). The aqueous phase is shown in blueish grey while the gas phase is white. The interface area is highlighted by the wave-like surfaces on the aqueous phase. **(B)** Radial distribution functions between the centres of mass of the proteins during the last 400 ns of the molecular dynamic's simulations at air-water interfaces of 8 trypsin molecules (red line), 8 Trypsin molecules + 72 HPB-LIKE (blue line), as well as 8 Trypsin molecules + 72 HP-LIKE (purple line). Probability density peaks at short protein-protein distances indicate aggregation, while a flat distribution would indicate no protein-protein interactions. The mean values with standard errors from 7 blocks are presented. .

As seen in Figure S8A, a trypsin by itself remains at the centre of the aqueous phase and does not concentrate on the surface. Notably, it still forms multimers, which is an indicator for aggregation. In the presence of HPB-LIKE (Figure S8A b), the cyclodextrins concentrate at the air-water interface. Due to the cyclodextrin-trypsin binding affinity, the trypsin proteins can be dragged by the cyclodextrins closer to the air-water interface, which might increase the risk of unfolding. HP-LIKE (Figure S8A c) also tends to concentrate at the air-water interface, and, due to the high affinity to both trypsin and itself, forms a compact aggregate that spans between the two air-water interfaces of the simulation. The radial distribution functions of the centres of mass of the trypsin proteins in the last 400 ns of the simulations at air-water interfaces are shown in Figure S8B. Compared to the radial distribution functions in bulk solution (Figure S7B), trypsin (red line) exhibits a higher first peak and an additional peak around 4.3 nm. Also, the position of the second peak moves to shorter distances (3.5 instead of 3.8 nm). The tail of the radial distribution

function is more uneven and exhibits lower probabilities. These observations indicate that the air-water interface region is energetically unfavourable for trypsin, which leads to more crowding in the bulk solution at the centre of the simulation box. Compared to the simulation with eight trypsin molecules, the addition of HPB-LIKE (blue line in Figure S8B) shifts the first peaks of the radial distribution function to longer distances by about 0.5 to 0.8 nm. This shift is not as pronounced as in Figure S8B, indicating that HPB-LIKE is not directly binding at the protein-protein binding sites. In addition, the peaks at short distances are significantly higher, which means more aggregation than in bulk solution. This can be explained by the propensity of HPB-LIKE to move to the air-water interface. Since many HPB-LIKE molecules are located at the interface, the effective concentration of HPB-LIKE in the bulk solution is lower. This means that fewer HPB-LIKE molecules will occupy the energetically less favourable docking position f of Figure S7A, which is responsible for preventing the protein-protein interactions a, b, c, and f of Figure 15. With more protein-protein binding modes being available, trypsin can form cross-networks under those conditions. In addition, the presence of HPB-LIKE leads to a more crowded environment, which forces the proteins to move closer together. Interestingly, the radial distribution function of HP-LIKE (purple line in Figure S8B) has its first peak at a longer distance than pure trypsin or trypsin with HPB-LIKE. This might be interpreted that HP-LIKE is more effective at preventing trypsin-trypsin aggregation at air-water interfaces. However, a comparison to the radial distribution function of HP-LIKE in bulk solution (purple line in Figure S7B) reveals that the positions of the first two peaks have remained almost the same. The first peak at the air-water interface is higher and broader than in bulk solution, while the second peak is lower. In addition, the third peak of the radial distribution function has moved to a shorter distance compared to the bulk solution (5.3nm instead of 6nm). This can probably be explained by Figure S8A c, where HP-LIKE concentrates at the air-water interface and, with its strong interactions with trypsin, forms a bridge to the HP-LIKE at the interface at the opposite end of the simulation box.

3. Discussion

3.1. Molecular Modeling of Trypsin and cyclodextrin systems

Molecular dynamics simulations of eight trypsin molecules revealed that there is a propensity to form stable multimers, which represent a pre-stage to aggregates. This propensity is higher at higher temperatures. The main trypsin-trypsin interactions were identified with protein-protein docking, showing that multiple pathways might lead to aggregation. These multiple, non-overlapping pathways obtained from the docking data imply that trypsin can potentially form multimers (Supplementary Figure S5). The molecular dynamics simulations revealed that HP not only has a strong binding affinity towards trypsin but also towards other HP molecules, which is attributed to the presence of three additional HP groups. As they form aggregates with themselves or trypsin, these aggregates are so compact that they might span between the interfaces. In other words, HP-LIKE CDs stick trypsin molecules together and tend to attach trypsin aggregates to the air-water interface. In this way, trypsin is destabilized even more by HP-LIKE CDs due to its preference to move towards the air-water interfaces and can drag trypsin molecules along to this interface. The molecular docking of HPB-LIKE and HP-LIKE CDs, identified which protein-ligand binding modes can potentially prevent protein aggregation, and which binding modes are energetically more favorable. Based on the relative binding affinities of the different binding poses and the capability to prevent certain dimerization pathways, this CD showed that its propensity to prevent trypsin from aggregation depends on its applied concentration. As air-water interfaces represent a hydrophobic environment, this might increase the probability of partial unfolding, which could be another cause of protein aggregation. However, HPB-LIKE CDs might be more effective at avoiding trypsin-trypsin aggregation at interfaces (Figure S8B).

References

(1) Kumar, V.; Errington, J. R. Application of the Interface Potential Approach to Calculate the Wetting Properties of a Water Model System. *Mol Simul* **2013**, *39* (14–15), 1143–1152. https://doi.org/10.1080/08927022.2013.817672.

(2) Van Gunsteren, W. F.; Berendsen, H. J. C. Algorithms for Macromolecular Dynamics and Constraint Dynamics. *Mol Phys* **1977**, *34* (5), 1311–1327. https://doi.org/10.1080/00268977700102571.

(3) Åqvist, J.; Wennerström, P.; Nervall, M.; Bjelic, S.; Brandsdal, B. O. Molecular Dynamics Simulations of Water and Biomolecules with a Monte Carlo Constant Pressure Algorithm. *Chem Phys Lett* **2004**, *384*, 288–294. https://doi.org/10.1016/j.cplett.2003.12.039.

(4) Steinbach, P. J.; Brooks, B. R. New Spherical-cutoff Methods for Long-range Forces in Macromolecular Simulation. *J Comput Chem* **1994**, *15* (7), 667–683. https://doi.org/10.1002/jcc.540150702.

(5) Darden, T.; York, D.; Pedersen, L. Particle Mesh Ewald: An N·log(N) Method for Ewald Sums in Large Systems. *J Chem Phys* **1993**, *98* (12), 10089–10092. https://doi.org/10.1063/1.464397.

(6) Roe, D. R.; Cheatham, T. E. PTRAJ and CPPTRAJ: Software for Processing and Analysis of Molecular Dynamics Trajectory Data. *J Chem Theory Comput* **2013**, *9* (7), 3084–3095. https://doi.org/10.1021/ct400341p.

(7) Roberts, C. J. Therapeutic Protein Aggregation: Mechanisms, Design, and Control. *Trends in Biotechnology*. Elsevier Ltd 2014, pp 372–380. https://doi.org/10.1016/j.tibtech.2014.05.005.
